# The interplay between *Trichuris* and the microbiota

**DOI:** 10.1017/S0031182021000834

**Published:** 2021-12

**Authors:** Melissa A. E. Lawson, Ian S. Roberts, Richard K. Grencis

**Affiliations:** 1 Lydia Becker Institute for Immunology and Inflammation, Manchester, M13 9PT, UK; 2 Wellcome Trust Centre for Cell Matrix Research, Manchester, M13 9PT, UK; 3 Division of Infection, Immunity and Respiratory Medicine, Manchester, M13 9PT, UK; 4 School of Biological Sciences, Faculty of Biology, Medicine and Health, Manchester Academic Health Science Centre, University of Manchester, Manchester, M13 9PL, UK

**Keywords:** Germ-free mice, microbial diversity hypothesis, microbiota, *Trichuris muris*

## Abstract

Parasitic worms are amongst the most common pathogens to infect humans and have a long-established history of inflicting disease in their hosts. There is a large body of evidence that states intestine-dwelling helminths ensure their survival by influencing the host immune response against them. In recent years, it has become apparent that the large and diverse microbial communities that exist in the gastrointestinal (GI) tract of the host and within the parasite itself have a pivotal role in worm survival and persistence. Using a variety of mouse models (including laboratory, germ-free and rewilded mice), there have been new insights into how bacteria and worms interact with each other; this includes the discovery that *Trichuris* is unable to hatch and/or infect their host in the absence of bacteria, and that these worms contain a *Trichuris*-specific gut microbiota. These interactions are determined in part by the capacity of the host, gut microbiota and worms to communicate *via* metabolites such as butyrate, which are microbially derived and have known immunoregulatory properties. By exploring the contribution of gut bacteria to worm infections and the intricate relationship that exists between them, an exciting and emerging field in whipworm parasitology is established.

## Graphical abstract

**Figure:**
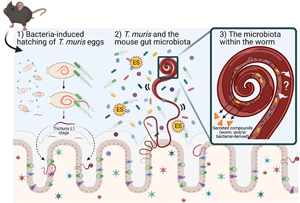

## Introduction

The accepted view of the gastrointestinal (GI) tract is an open tube from the mouth to the anus that has evolved to control and interact with the external environment. The host GI tract is the largest immunological organ in the body; involving mechanisms to identify, control and eliminate dangerous pathogens present, while at the same time minimizing inappropriate inflammatory responses against benign antigens such as commensal bacteria and diet. In addition, the mammalian host GI tract also provides a favourable ecosystem with a stable temperature of approximately 37°C and a continual influx of nutrients that permit the growth of a complex microbiota. While numerically bacteria are the largest inhabitants of the GI tract, the GI tract has a number of diverse macro- and microbiotic communities which include viruses, fungi, protozoans and of particular interest for this review intestinal-dwelling parasitic nematodes.

Almost every mucosal surface on a mammal's body is heavily colonized with a variety of bacteria. Colonization begins at birth and initiates a variety of complex processes that lead to the establishment of a truly symbiotic relationship between the microbiota and host. We are only beginning to understand the numerous roles the microbiota plays in human health and the development, progression and resolution of disease. The gut microbiota (bacteria that inhabit the GI tract) has been the focus of intense research for the last few decades and is known to contribute to immunity, host metabolism and behaviour. The microbiota is known to mediate these functions by both direct (cell-to-cell) and indirect (bacterial-derived by-products) mechanisms which can involve interactions with the mammalian host immune cells during development (Elahi *et al*., 2013; Gensollen *et al*., 2016), hormone and behaviour signalling in the endocrine system (Lyte and Bailey, 1997; Diaz Heijtz *et al*., 2011), influencing disease progression of autoimmune disease such as inflammatory bowel disease (IBD), (Halfvarson *et al*., 2017) or type 1 diabetes (Markle *et al*., 2013), as well as affecting drug metabolism and efficacy (Zimmermann *et al*., 2019). From the time of birth, there is a known bidirectional interplay between the mammalian host and microbiota that is influenced by the composition, in terms of both diversity and abundance, of the gut microbiota which impacts host health (Dominguez-Bello *et al*., 2010).

While gut microbiota coevolves with the host immune system to influence host health, key factors that influence this process include enhanced sanitation and antibiotic usage, which are different in low/middle-income countries compared to high-income countries. Epidemiological evidence indicates that in high-income countries, there is low to no incidence of enteric parasite infections, but there has been a steady rise in autoimmune and allergic-related conditions, like asthma, eczema, diabetes and IBD in humans (Bach, 2002). Improved diagnostic tools to identify the incidence and prevalence of disease have likely contributed to the rise in autoimmune and allergic-related conditions observed over time; however, it is also well documented that impeding parasite exposure (and the subsequent effects of parasitic infections) in early life results in a disruption of the normal immune development and leads to a greater predisposition to autoimmune and allergic diseases in life (Strachan, 1989; Bach, 2002). This theory is called the ‘hygiene hypothesis’ (Strachan, 1989; Bach, 2002), and has been further refined to the ‘microbial diversity’ hypothesis (also known as the ‘old friends’ hypothesis or ‘microflora hypothesis’) which proposes that enhanced sanitation and hygiene in high-income countries has resulted in a reduction of microbial exposure and/or gut microbiota composition in early life, that alters immune development and causes a predisposition to allergic diseases (Wold, 1998; Rook, 2009; Scudellari, 2017). Therefore, the means by which the gut microbiota can impact human health is highly complex and varied, and there is growing evidence of epidemiological, microbiological and immunological data that support these hypotheses. However, there tends to be a focus on research that examines the bidirectional relationship between the microbiota and host. The presence of enteric multicellular parasites adds an additional level of complexity, and increases our understanding of the pervasive importance of the gut microbiota, and how these relationships impact mammalian host health and disease. Here we will focus on the interaction between the gut microbiota and enteric parasites, specifically whipworm infections by *Trichuris* and its dependency on the gut microbiota for propagation, persistent infection and survival in the mammalian gut.

## The gut microbiota

The gut microbiota is a highly dynamic community of >1000 different bacterial species primarily comprised of phyla belonging to Firmicutes, Bacteroidetes, Actinobacteria, Proteobacteria, Fusobacteria and Verrucomicrobia (Arumugam *et al*., 2011). In return for the host providing a protective, nutrient-rich niche for the gut microbiota to inhabit, some of these bacteria perform essential functions for host health including the synthesis of vitamins (such as vitamin K, Gustafsson, 1959), the degradation of complex nutrients (Bäckhed *et al*., 2005), limiting pathogen invasion (Stecher *et al*., 2005) and contributing to immune system development (Stappenbeck *et al*., 2002; Flint *et al*., 2012). During the first three years of life, there is a succession of colonization events, for humans, this is marked by events such as a diet change from milk to solid foods that promotes the development of a stable gut microbial composition that is able to contribute to host health and readily adapt to lifestyle changes over a life time (Wu *et al*., 2011). Antibiotic usage is the most robust and quick-acting factor that can alter microbial diversity, closely followed by microbial changes induced by changes to the host diet (Jakobsson *et al*., 2010; David *et al*., 2013). An imbalance in the microbial community (referred to as a state of dysbiosis) can have a profound impact on increasing host susceptibility to disease and it is typically associated with an increase of facultative anaerobic bacteria (Shin *et al*., 2015; Rizzatti *et al*., 2017).

Technological advancements in the ability to decipher the composition and function of the microbiota, including targeted culturing techniques for recalcitrant strains, culture-independent techniques including 16S rRNA amplicon sequencing and whole-genome sequencing coupled with metabolomics and computational analysis have transformed our understanding of how important the gut microbiota is in host health (Browne *et al*., 2016). Bacterial composition of the microbiota, in particular microbial diversity, is crucial, as it increases the likelihood of containing species that are capable of degrading complex carbohydrates into microbial fermentation products being present in the GI tract. The gut microbiota is dominated by obligate anaerobes, specifically the phyla Firmicutes (Gram-positive) and Bacteroides (Gram-negative). Members of these phyla are characterized by encoding a large variety of enzymes that hydrolyse complex dietary and host carbohydrates that provide benefit to the host (Flint *et al*., 2012; El Kaoutari *et al*., 2013). For example, short-chain fatty acids (SCFAs) are small organic carboxylic acids (with chain lengths up to six carbons), of which acetate (C2), propionate (C3) and butyrate (C4) are the most abundant metabolites produced from anaerobic fermentation of dietary fibre and resistant starch. There is growing evidence that SCFA function as metabolic moieties in crosstalk between the host and gut microbiota to exert immunomodulatory activity upon the host. Namely these metabolites have also been identified as an energy supply for colonocytes, regulation of T regulatory (Treg) cell populations in the mucosal immune system and a potential role for treatment of autoimmune diseases (Cummings *et al*., 1987; Furusawa *et al*., 2013; Gill *et al*., 2018).

## Germ-free animal models and *Trichuris*

Germ-free animals do not contain any detectable microorganisms, and are commonly used as a reductionist approach to investigating the relationship between the host and single (or known multiple-gnotobiotic) bacterial species (Smith *et al*., 2007; Flint *et al*., 2012). Recently these approaches have been used to triangulate the relationships between the host, its microbiota and intestinal multicellular organisms (such as helminths).

In the late 19th century, Louis Pasteur was the first to postulate that bacteria residing in the GI tract formed a relationship with the host that was critical for survival (Pasteur, 1885). This theory was perceived to be true, until a decade later it was disproved by raising germ-free guinea-pigs, and demonstrating life was possible without bacteria (Nuttal and Thierfelder, 1895). However, throughout the early 20th century, scientists continued to pursue the understanding of the relationship between the host and the bacteria that inhabit the GI tract. Using a variety of animal models and human subjects, researchers found that bacteria must be confined to mucosal surfaces for health (including areas such as the intestinal lumen, nasal cavity and aspects of the reproductive system), and a breach of this containment resulted in severe sepsis and/or death (Cushing and Livingood, 1900; Reyniers, 1959). Today, germ-free mice are the most common germ-free model used due to the number of immune-comprised models available, and their ability to breed frequently with large litters in a single cage. Germ-free mice are born and raised in sterile flexible-film isolators maintained under positive pressure to the external environment, and receive sterile chow, bedding, water and HEPA-filtered sterile air to limit exposure to live microbial antigens (Smith *et al*., 2007). Interaction and manipulation of the gut microbiota by enteric worms as a potent mechanism to regulate the host immune system and worm persistence are emerging as a topic of study where the use of germ-free models can provide us insight into this intricate and complex relationship. For intestinal parasitic infections, such as *Trichuris*, there is a critical dependency on a living host for propagation and survival, making germ-free models a means to provide mechanistic insight into the relationship between bacteria and worm infection (Hayes *et al*., 2010; Leung *et al*., 2018; White *et al*., 2018).

## Helminth-microbe interactions

### Microbial cues to induce *Trichuris muris* infection

*Trichuris trichiura* is one of the most prevalent soil-transmitted parasites, and infects approximately 500 million people worldwide (World Health Organization, 2020). Although whipworm infections rarely cause mortality in humans, individuals with a high worm burden can suffer from severe morbidities including diarrhoea/abdominal pain, malnutrition and infected children often have impaired growth/physical development that impacts their health status for life (World Health Organization, 2020). Bacterial diversity and density vary along the GI tract, with the densest microbial population residing within the caecum and colon of both mice and humans, reaching upwards of 100 trillion bacteria. Intestinal whipworms also inhabit this microbial-dense niche, and it is highly likely that these worms and the surrounding microbiota interact with each other (either directly or indirectly), as well as their mammalian host. An overview of how *Trichuris* interacts with the mammalian host, in particular the host immune system during acute and/or chronic infection, and the details of the *T*r*ichuris* life cycle is presented in this special issue by Mair *et al.* (2021).

To decipher how bacteria in the human GI tract interacts with *T. trichiura*, researchers use mouse models as the host, and infect mice with the murine-specific *T. muris* whipworm. *Trichuris muris* infection follows the same strict oral-faecal route of infection, and worms embed themselves in mouse caecum and colon tissue, which closely mimics human whipworm infection and pathology (Klementowicz *et al*., 2012). As with *T. trichiura*, *T. muris* remain within the large intestine (which includes the caecum and colon) for their lifespan, and this has resulted in a complex relationship between the parasite and mouse microbiota, where it is known that the parasite manipulates and exploits the gut bacteria to induce persistent infection (White *et al*., 2018). It is now apparent that *T. muris* is dependent on cues from both the host and gut microbiota to initiate infection and generate pathology in the host. Embryonated eggs incubated with gut explants containing caecal bacteria, or bacterial cultures of *E. coli* (Hayes *et al*., 2010) and/or *Staphylococcus aureus* (Koyama, 2013) were shown to provide important microbial cues that enable successful worm larvae (stage one, L1) hatching to occur at the start of infection. Hayes *et al*. (2010) also found that the intestinal bacteria formed aggregates around egg poles, and that this interaction was mediated by adhesion organelles on the surface of bacterial cell membranes, namely type 1 fimbria. Hatching of embryonated eggs from the closely related whipworm *T. suis* (pig trichuriasis) did not occur using the same bacterial species (both Gram-negative *Escherichia coli* strains and Gram-positive *Lactobacilli*, *Streptococci* and *Enterococci* strains) that induce *T. muris* hatching (Vejzagić *et al*., 2015), suggesting that host–worm–microbiota specific interactions exist. Antibiotic treatment prior to *T. muris* infection resulted in a significant depletion of gut microbiota that reduced hatching rates, further demonstrating a dependence of *Trichuris* on the microbiota to initiate infection (Hayes *et al*., 2010).

### The interaction of *Trichuris muris* and the gut microbiota

**Fig. 1.**
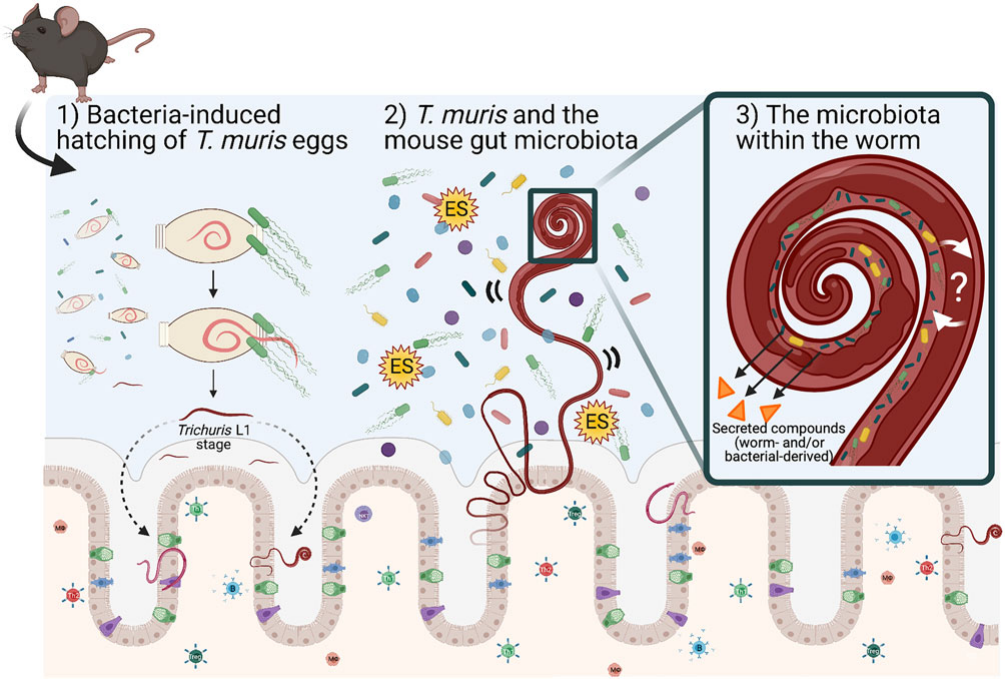
Schematic representing known and proposed mechanisms of how *T. muris* interacts with the large intestinal microbiota. (1) Upon entry into the large intestine, worm eggs interact with bacteria to promote hatching and initiate infection of the large intestine. (2) Once embedded in host epithelium, *T. muris* is proposed to influence the composition of the mouse gut microbiota by direct and indirect mechanisms (competition for space and/or nutrients for growth, by the secretion of metabolites including *Trichuris* ES and bacterial-derived compounds). (3) *T. muris* has also evolved to have its own worm gut microbiota, which is derived from the mouse gut microbiota. The identity and function of worm-selective pressures on their gut microbiota, and how the worm-specific bacteria interact with the worm host are unknown. Schematic shows the gut epithelial barrier with epithelial cells (beige), Goblet cells (green), Tuft cells (blue) and enteroendocrine cells (purple) covered in thick mucus (grey). Image created with BioRender.com.

Many enteric parasites have a tropism for the large intestine, which is the same mucosal niche as the majority of bacteria in the gut microbiota. Within this niche, *Trichuris* and the gut microbiota are likely to interact with each other through either direct or indirect mechanisms, and it is this interaction and relationship that can influence each other's growth and survival in the mammalian GI tract. *Trichuris muris* infection protects immunodeficient mice (*Nod2−/−*) from intestinal abnormalities associated with this IBD-mouse model by enhancing the growth of protective bacterial populations like the Clostridiales, and in turn inhibiting pro-inflammatory species (Ramanan *et al*., 2016). What mechanisms are responsible for inducing changes in the mouse gut microbiota composition (in terms of abundance and diversity) and how they subsequently induce anti-inflammatory responses in the host are largely unknown. We propose mechanisms for altering the gut microbiota may include the physical presence of whipworms in the lumen (worms embedded in intestinal epithelial cells may disrupt surface cell adhesions for bacteria and possible competition for space), secreted worm-derived compounds, competition for nutrients against members of the gut microbiota, and/or worm-induced changes in the host during infection that alter the local GI immune (Th1 or Th2) environment that may also affect the survival of members within the gut microbiota (Fig. 1).

In the absence of all microorganisms, as observed in germ-free mice, a genetically susceptible host becomes resistant to *T. muris* infection using either embryonated eggs or *in vitro* hatched L1 larvae (White *et al*., 2018). On the other end of the spectrum, laboratory mice rewilded into the environment develop a more diverse microbiota which renders them more susceptible to *T. muris* infection with greater parasite burdens and worm biomass than their laboratory-dwelling counterparts (Leung *et al*., 2018).

Another study of rewilded mice found that the gut microbiota of *T. muris*-infected rewilded mice promoted the expansion of Firmicutes, Bacteroidetes, Proteobacteria phyla, and this compositional change was associated with a reduction in colonization resistance against other invading species (specifically members from the Enterobacteriaceae and Rikenellaceace families) when compared to rewilded uninfected mice (Bär *et al*., 2020). These findings provide further evidence of how important the microbiota is to whipworm infection, and how delicate the balance that exists between microbial diversity (in terms of both composition and abundance) and the severity and persistence for successful worm infection.

Using a well-established model of chronic worm infection, when laboratory wild-type mice are given a single low dose of embryonated eggs (20–30 *T. muris* eggs) upon transition from larvae to adult worms (day 28 post infection), the gut microbiota undergo compositional changes (Houlden *et al*., 2015; Schachter *et al*., 2020). Specifically, bacterial taxa like *Firmicutes*, *Mucispirillum* and *Proteobacteria* increase as a result of infection; whilst *Parabacteroides* and *Prevotella* decrease in abundance (Holm *et al*., 2015; Houlden *et al*., 2015; Schachter *et al*., 2020). The observed changes in microbial composition from infection appear to be parasite-dependent and transient in nature. In addition, this altered gut environment makes the host less favourable for re-infection with *Trichuris* (White *et al*., 2018), but whether or not it makes the microbiota more permissive to other infections, like *Salmonella* (as observed with infection of the small intestinal parasite *Heligmosomoides polygyrus bakerii*), has yet to be studied (Brosschot *et al*., 2021). However, three months post infection when no mature whipworms are detectable, the gut microbiota regains bacterial diversity equivalent to their uninfected counterparts (Holm *et al*., 2015; Houlden *et al*., 2015).

We know that the absence of a microbiota (germ-free mice) prevents *Trichuris* hatching and infection, whilst the presence of a highly diverse microbiota actually enhances host susceptibility to infection. However, more research is needed to understand how microbial diversity influences worm persistence and survival in the gut, and what role the microbiota may have in manipulating the host immune system response against the invading worms. It is well documented that susceptibility to worm infection is largely dictated by the host immune system response against parasitic infection; CD4+ T helper cell 2 (Th2) responses mount a targeted immune response which leads to rapid worm expulsion, whereas if the host mounts a CD4+ T helper cell 1 response against whipworm infection, the host experiences chronic infections with low worm burdens (Else *et al*., 1992). This is in contrast to other helminth infections where parasite-derived ES products activate tuft cells to initiate Th2 responses, and specifically tuft cell hyperplasia in the gut during chronic infections (Gerbe *et al*., 2016). Germ-free data from enteric roundworm infection using either antibiotic-treated of L3 *H. polygyrus* larvae or with eggs hatched using an auxotrophic *E. coli* strain (HA107 that cannot replicate in mice without exogenous supplementation of microbial amino acids) found that worms had impaired fitness as noted by reduced fecundity, and the germ-free hosts had mounted a more robust type 2 humoral responses in the absence of gut bacteria (Rausch *et al*., 2018; Russell *et al*., 2020) implicating a possible function of the microbiota in buffering host immune responses to worm infections. Mouse models with varying degrees of microbial diversity (from rewilded to single species colonization of germ-free mice) highlight how critical the composition of the gut microbiota is for *Trichuris* infection. Manipulation of the gut microbiota in mice to contain genetically altered bacteria may shed light on how these bacteria interact with *Trichuris* and what functions different genera of bacteria have that contribute to successful whipworm infection.

### Reoccurring Trichuriasis and the gut microbiota

A limited number of field studies have been performed investigating the relationship between intestinal helminth infections and the host microbiota; although there are conflicting reports. A field study examining the microbiota composition of Ecuadorian children found *T. trichuria* infection did not induce significant changes in the gut microbiota composition (Cooper *et al*., 2013), whereas individuals infected with intestinal helminths (including *Trichuris*) within the Malaysian indigenous (Orang Asli) populations were found to have higher microbial diversity than their negative counterparts (Lee *et al*., 2014). It is possible that the conflicting data in human field studies, and their comparison to mouse models are due to the fact that infected individuals are rarely exposed to a single, low-dose helminth infection but rather continuous re-occurring infection by the same or multiple parasites. A recent study in Tanzania, was one of the first to identify a reduction in the abundance of gut bacteria: Bacteroidetes, Proteobacteria and Actinobacteria associated with a cohort of women and children solely infected with *T. trichuria* (Chen *et al*., 2021); which is similar to the microbial changes reported in mouse studies (Holm *et al*., 2015; Houlden *et al*., 2015). Parallels between human and mouse studies were further addressed by identifying bacterial taxa that increased in both *T. trichuria*-only infected Indonesian individuals and *T. muris*-infected mice, these genera included *Bacteroides*, *Collinsella*, *Subdoligranulum*, *Escherichia*/*Shigella*, *Prevetoella* and *Streptococcus* (Rosa *et al*., 2021). There has also been the development of a mouse model to partially address the discrepancy in human and mouse data that involves a trickle model of *T. muris* infection, where mice are routinely (weekly) infected with low doses of embryonated eggs, thereby closely mimicking the low-dose chronic human infection by *T. trichiura* (Glover *et al*., 2019). This simulated model of natural infection with repeated exposure to *T. muris* caused a decrease in microbial diversity early on during infection that rapidly resolved over time to control levels upon repeated exposure (Glover *et al*., 2019). Interestingly, this tickle model was associated with the development of partial protective immunity supporting the concept of a complex interplay between host immunity, parasite and microbiota. Overall, laboratory experiments and field studies indicate that primary infection by *Trichuris* influences the microbiota composition, but the true nature of the relationship between the microbiota and intestinal parasites remains largely unknown.

### *Trichuris muris*’ intimate relationship with bacteria

While multiple studies suggest that the large metazoan parasite *Trichuris* has evolved to respond to microbial cues in the gut to initiate hatching (and infection) of the host, mature whipworms have also been shown to harbour their own unique gut microbiota by 16S rRNA gene denaturing gel electrophoresis and fluorescence *in situ* hybridization (White *et al*., 2018). Irrespective of the host microbiota composition, mature whipworms acquire a distinct microbiota selected from members residing in the host gut during infection (White *et al*., 2018). The worm microbiota is largely dominated by bacterial species belonging to the phylum *Proteobacteria* (White *et al*., 2018), which begs the questions as to how does the worm select these bacteria, why *Proteobacteria* and what role do these particular bacteria provide for worms? Currently, worm-specific selective pressures on bacteria are unknown, and whether the bacteria are selected based on their function in the gut microbiota, their ability to adapt to new environments (such as the worm gut) or if they are exploited as an energy source (food) still need to be answered. The fact whipworms evolve and generate their own microbiota adds complexity to the host–parasite–microbiota relationship and raises new questions in understanding each of the mutualistic relationships present in the gut. Future work may include using a defined model system of a single microbial species in a germ-free host with the nematode *Trichuris* to address these outstanding questions, simply because of the many advantages this system has to offer in terms of experimental tractability for exploring the relationship between *Trichuris* and the microbiota.

## Metabolites as moieties for cross-talk

*Trichuris* has developed effective strategies against the host immune system by exploiting its immunoregulatory properties to permit chronic infection; identification of potential candidates in this cross-talk is likely to be metabolites produced by (directly or indirectly) the worm during infection. In chronic infection, *T. muris* worms reach maturity at day 33 post infection, at which point mucosal immune and pathological changes in response to infection have occurred, and the microbial population and the worm have adapted to each other. Faecal metabolite analysis at day 41 of chronic infection identified increased concentrations of amino acids including phenylalanine, serine, threonine, leucine, ornithine and glycine, with a decrease in the abundance of vitamin D2/D3 derivatives, fatty acid and glycerophospholipid metabolites, compounds involved in heme degradation and metabolites involved in the biosynthesis of tryptophan, lysine, cystine and arginine amino acids when compared to naïve samples (Houlden *et al*., 2015). This altered metabolic profile can be interpreted as a hallmark of the skewed gut microbiota due to chronic infection and the ability of the microbiota to differentially harvest resources from the host diet. What is currently unclear is the origin of these observed metabolite changes and the relative contributions of the host, parasite and microbiota.

A reduction in luminal SCFA butyrate occurs during infection, and in turn host cells downregulate the expression of butyrate transporters, suggesting a transition to utilising other readily available energy sources available in the gut (White, 2016). Bacteria are the major source of SCFA in the gut, specifically the abundance and function of butyrate producers including taxa within the Firmicutes phylum like *Clostridiales* clusters IV and XIVa, and *Ruminococcaceae* (Louis *et al*., 2010), as well as members of Bacteroidetes, Actinobacteria and Proteobacteria that produce and utilize butyrate for the synthesis of essential amino acids are able to influence host health. Many of these bacteria are reduced in number during worm infection, and contribute to the alterations of microbial-derived metabolites like butyrate (Houlden *et al*., 2015). *Trichuris* is unable to synthesize butyrate, and supplementation of butyrate during chronic infection had minimal impact on worm burden or disease severity (Colombo and Grencis, unpublished data). Interpretation of this data may suggest that the worm presence and infection in the large intestine induces a mucosal inflammatory response that makes the gut less favourable for the growth of butyrate-producing bacteria like *Clostridia* and *Ruminococcacae* that accounts for the reduction in butyrate-regulated FoxP3+ CD4+ Treg cells in the colonic lamina propria (Houlden *et al*., 2015).

### *Trichuris*-specific metabolites

*Trichuris muris* eggs are devoid of bacteria (White *et al*., 2018); therefore, at this life cycle stage, all identified metabolites are likely to be worm origin (rather than microbial). Using mass spectrometry, *T. muris*-egg specific metabolites were identified to belong to amino acid metabolism, carbohydrate metabolism and nucleotide metabolism (Yeshi *et al*., 2020). Within 90 min of entering the gut, the first-stage larvae are released from the egg and begin to borough into the epithelium; at which point the worms begin to grow and mature and release proteins into the niche called excretory–secretory (ES) products (Bancroft *et al*., 2019). In contrast to other parasitic metabolites, such as those produced by *Nippostrongylus brasiliensis* infection; *Trichuris* ES-enriched fractions contain an abundance of fatty acids including SCFA, amino acids and enriched organic compounds (Wangchuk *et al*., 2019). Specifically, lactic acid, oleic acid, succinate acid, the SCFAs: propionate, butyrate and acetate; alanine, glutamine, sugars: fructose, galactose, glucosamine and glucose-6-phosphate; and adenine and uridine (nucleobases/nucleosides) were found to be increased (Wangchuk *et al*., 2019). However, *T. muris* lacks known gene homologs for the synthesis of these metabolites, for example, there are no gene clusters identified to generate *de novo* synthesis of SCFA butyrate in the worm genome (White, 2016). Therefore, the identified metabolites by Wangchuk *et al*. (2019) are more likely to be a combination of *Trichuris-*derived metabolites and metabolic compounds generated by their own gut microbiota, which adult worms acquire from the host microbiota.

In chronic infection, the immune system generates a ‘tolerogenic-like’ immune response against the worms, which is likely to be mediated from a number of *Trichuris*-specific anti-inflammatory metabolites increased in ES-fractions including glucosamine, uridine and butyrate (Wangchuk *et al*., 2019). High levels of succinate and lactic acid were found in the ES-fraction, and the immunoregulatory effect of butyrate, succinate and lactic acid may have a role in contributing to anaerobic metabolism in the caecum, which would reflect a possible beneficial trait of the worm gut microbiota (Wangchuk *et al*., 2019). Extracellular vesicles (EV) also present in the ES faction of *T. muris* are internalized by the host cells and been found to contain a multitude of proteins including those involved in proteolysis, mRNAs and microRNAs (Eichenberger *et al*., 2018). Recently, using mouse caecaloids (organoids made from the caecum), it has been shown that *T. muris*-specific EVs downregulate responses to nucleic acid recognition and type 1 interferon immune-mediated responses (Duque-Corre *et al*., 2020). How worm-specific EVs interact with the microbiota is unclear (Eichenberger *et al*., 2018). Not only does the identification of *T. muris-*secreted metabolites during infection provide a tool for understanding how whipworms interact with both the host and bacterial environment, but also may lead to the design of novel approaches to controlling chronic worm infections.

## Treatments

A variety of anthelmintic drugs are used to treat *Trichuris* infections in humans and livestock. For *T. muris* infection anthelmintic drugs include, but are not limited to the administration of albendazole, mebendazole, levamisole, pyrantel pamoate and ivermectin that are also commonly used to treat *T. trichiura* infections (Keiser *et al*., 2012). How these drugs impact the gut microbiota in the host and worm remains largely elusive. Houlden *et al*. (2015) found mebendazole treatment on naïve mice did not influence the composition of the gut microbiota, however many of the other drugs have yet to be studied. A reduction in microbial species diversity was observed in antibiotic-treated mice prior to infection that led to both reduced worm burden in the mice, and a modulated T-cell-dependent immune response mounted against existing worms (Hayes *et al*., 2010; Houlden *et al*., 2015). Transient antibiotic treatment of mice prior to a high-dose *T. muris* infection led to enhanced susceptibility to infection (Scott *et al*., 2018) and it was proposed that this was dependent upon the generation of dysregulated macrophages promoting type 1 immune responses, again supporting a complex interplay between host and microbiota influencing infection outcome. As an alternative to drug therapy, repeated administration of live beneficial bacteria like the probiotic strain *Lactobacillus rhamnosus* (isolate JB-1) caused an acceleration of worm expulsion in mice, mediated by an IL-10/Goblet cell pathway induced in the murine host (McClemens *et al*., 2013). Manipulation of the host microbiota with probiotics to reduce worm burden further highlights the intricate relationship between the gut microbiota and whipworms during infection.

In humans, there is limited and conflicting information regarding the impact of anthelmintic treatment on the gut microbiota. The majority of infected individuals have worm burdens below clinical thresholds, and that can remain in their host for years contributing to poor nutrition and impaired growth and development, particularly in children (Callender *et al*., 1992; Nokes *et al*., 1992). It is unclear if the enhanced burden of chronic whipworms or the altered gut microbiota associated with these infections is the cause or effect of these health concerns in individuals, and if employing long-term administration of prebiotic and/or probiotic treatments to alter the gut microbiota would be a step forward in controlling parasitic infections.

## Conclusions

The importance of the microbiota during helminth infection is becoming increasingly recognized, and is also the start of an exciting and crucial area of parasitological research. Microbial changes have been reported for multiple intestinal helminths including infections with *Ascaris lumbricoides* (Ramírez-Carrillo *et al*., 2020), *H. polygyrus* (Walk *et al*., 2010; Kreisinger *et al.*, 2015), *Necator americanus* (hookworm; Cantacessi *et al*., 2014) and of interest for this review *T. trichiura* (Lee *et al*., 2014; Chen *et al*., 2021) and *T. muris* (Holm *et al*., 2015; Houlden *et al*., 2015; Schachter *et al*., 2020).

Using novel tools such as germ-free models to decipher the intricate relationship that the microbiota has with whipworms opens new perspectives on how these helminths function and infect their hosts. In addition, field studies of re-wilding mice, or potentially wild, naturally occurring infection mouse models provide evidence into the complexity and dependence of the gut microbiota and *T. muris*, and possible insight into *T. trichuria* infections. Helminth metabolites are also likely to be candidates for regulating the immune system, positively contributing to microbial richness and diversity in individuals who suffer from chronic inflammatory diseases, or in turn as potential candidates for anthelminthic drugs (as discussed in Garcia-Bustos *et al*., 2019 for *Caenorhabditis elegans*). Harnessing how enteric parasites, such as whipworms, interact and modulate the gut microbiota will open new avenues for exploring anthelminthic treatments and addressing the growing concern of the ‘microbial diversity’ hypothesis regarding the dramatic increase in the incidence of allergic and autoimmune diseases in developed countries. Human trials using *Trichuris* eggs (specifically *T. suis*) show promise as a probiotic agent, and has been used in trials as a treatment for multiple sclerosis (Fleming *et al*., 2011, 2019; Yordanova *et al*., 2021; Williams *et al*., 2017). More recently, clinical intervention trials using *T. suis* eggs against psoriasis and IBD are ongoing in North America, and it will be interesting to see how *Trichuris* can reduce symptoms mediated by an altered immune response and potentially by changes in gut microbial composition (Prosberg and Petersen, 2018–2021). Understanding how *Trichuris* interacts with the gut microbiota during infection is of particular interest, as it is the organ that harbours the largest and most diverse microbial community within our bodies. In conclusion, we anticipate there will be substantial growth in this field and more research examining how to harness this information to develop new helminth therapies that control the spread and infection in humans.
